# Tissue-Resident Lymphocytes: Implications in Immunotherapy for Hepatocellular Carcinoma

**DOI:** 10.3390/ijms22010232

**Published:** 2020-12-28

**Authors:** Ji Won Han, Seung Kew Yoon

**Affiliations:** 1 Graduate School of Medical Science and Engineering, Korea Advanced Institute of Science and Technology, Daejeon 34141, Korea; tmznjf@catholic.ac.kr; 2 Division of Hepatology, Department of Internal Medicine, College of Medicine, The Catholic University of Korea, Seoul St. Mary’s Hospital, Seoul 06591, Korea; 3 The Catholic University Liver Research Center, College of Medicine, The Catholic University of Korea, Seoul 06591, Korea

**Keywords:** tissue-resident lymphocytes, hepatocellular carcinoma, immunotherapy

## Abstract

Hepatocellular carcinoma (HCC) is a hard-to-treat cancer. The recent introduction of immune checkpoint inhibitors (ICIs) provided viable options to treat HCC, but the response rate is currently not sufficient. Thus, a better understanding of ICI-responding cells within tumors is needed to improve outcomes of ICI treatment in HCC. Recently, tissue-resident memory T (T_RM_) cells were defined as a subset of the memory T cell population; this cell population is actively under investigation to elucidate its role in anti-tumor immunity. In addition, the role of other tissue-resident populations such as tissue resident regulatory T (Treg) cells, mucosal associated invariant T (MAIT) cells, γδ T cells, and invariant natural killer T (iNKT) cells in anti-tumor immunity is also actively being investigated. However, there is no study that summarizes recent studies and discusses future perspectives in terms of tissue resident lymphocytes in HCC. In this review, we summarize key features of tissue-resident lymphocytes and their role in the anti-tumor immunity. Additionally, we review recent studies regarding the characteristics of tissue-resident lymphocytes in HCC and their role in ICI treatment and other immunotherapeutic strategies.

## 1. Introduction

Hepatocellular carcinoma (HCC) is the fourth leading cause of cancer-related deaths in the world. In advanced-stage HCC, which cannot be treated by surgical resection, liver transplantation, local ablation, or transarterial chemoembolization (TACE), sorafenib has been the only treatment option for decades, although with limited efficacy. The recent development of immune checkpoint inhibitors (ICIs) for cancers such as melanoma and lung cancer have provided additional options to treat HCC, and anti-programmed cell death protein-1 (PD-1) agents such as nivolumab and pembrolizumab have been approved by the Food and Drug Administration (FDA) as second-line treatments after sorafenib based on phase I/II clinical trial results [1,2] with an approximately 20% objective response rate. However, a substantial proportion of patients does not respond to ICIs, and a recent nivolumab (CheckMate-459) phase III trial failed to reach its primary endpoint [3].

To achieve better efficacy of ICIs in HCC, robust biomarkers predicting response or determining if ICIs in combination with other therapeutic agents is beneficial need to be identified. In addition, elucidating the characteristics of CD8+ and CD4+ T cells, which are located in tumors and surrounding tissues, is also important, because they are major effectors that respond to ICIs and kill tumor cells. Recently, tissue-resident memory T cells (T_RM_) were identified; they are located and persist in the peripheral non-lymphoid tissues and react robustly and rapidly upon local antigen stimulation [4,5,6]. Their role in the anti-tumor immunity is currently being investigated [7]. Furthermore, other tissue-infiltrating lymphocytes such as tissue resident regulatory T (Treg) cells, mucosal associated invariant T (MAIT) cells, γδ T cells, and invariant natural killer T (iNKT) cells also have tissue-resident properties, and their role in anti-tumor immunity is also actively being investigated [8]. Substantial studies have revealed that tumor-infiltrating lymphocytes (TILs) may be potential targets of immunotherapeutic agents for HCC. A previous report showed that the presence of T cells and cytotoxic cells within TILs was associated with favorable patients’ survival in HCC [9]. Nevertheless, there is no study that summarizes recent studies and discusses future perspectives in terms of the characteristics of tissue resident lymphocytes, and their role in HCC immunotherapy. In this review, we discuss recent studies of tissue-resident lymphocytes in HCC and strategies to treat HCC.

A recent report revealed that tumor- and liver-infiltrating lymphocytes express CD69 over 50%, which is a hallmark of tissue-resident lymphocytes, in HCC patients [10]. These tissue-resident lymphocytes within the liver and tumor have unique characteristics in terms of their functions, phenotypes, and transcriptional properties, compared to the circulating or CD69 lymphocytes [11]. Therefore, we will review their unique characteristics and discuss that these unique properties are the possible targets for the novel immunotherapies for HCC. Furthermore, their characteristics seem to correlate with the histopathologic profiles of HCC and might be changed by ICI treatment [10], although this phenomenon needs to be validated in future large-scale clinical trials. The evaluation of tumor- or liver-infiltrating lymphocytes before and after ICI treatments could provide important information on their efficacy, and the present review might have a role as a guide for those analyses.

## 2. General Features of T_RM_ Cells

T_RM_ cells are a recently identified population of memory T cells. They reside in peripheral tissues, do not recirculate, and provide rapid and robust responses to local antigen stimulation. Generally, this population is composed of CD8+ T cells. Additionally, CD4+ T cells; FoxP3+ Treg cells; and innate-like T cells such as γδ T cells, NKT cells, and MAIT cells are broadly considered as part of the T_RM_ cell population. They commonly express CD69 and downregulate *S1PR1*, which prevents these cells from egressing out of the peripheral tissues [5,6,12]. CD103 is considered a representative marker of T_RM_ cells and is an integrin alpha E molecule that binds integrin beta 7, resulting in retention in peripheral tissues [5,6,13], although its expression is dependent on the type of tissues. For example, murine liver CD8+ T_RM_ cells do not express CD103, although they play a crucial role in protecting from liver-stage malaria infection [14]. In humans, a very small portion of CD69+ CD8+ T cells express CD103 (≈5%), which might be considered bona fide T_RM_ cells [15]. Recently, human liver CD69+ CD103− CD8+ T cells were reported to have tissue-resident phenotypes [16].

Peripheral blood T cells lack CD69 and CD103, which are considered traditional hallmarks of T_RM_ cells. In addition to these markers, CD49a [17] and C-X-C chemokine receptor type 6 (CXCR6) [18] are also considered as phenotypes of tissue residency. Importantly, T_RM_ cells do not express C-C chemokine receptor type 7 (CCR7) and CD62L, which are both expressed in naïve and circulating memory T cells [19]. Thus, T_RM_ cells have distinct phenotypes from circulating T cells (T_CIRC_).

Transcription factors such as *Hobit* and *Blimp1* are central regulators of T_RM_ development and maintenance in murine CD8+ [20] and CD4+ T cells [21]. In addition, the upregulation of *Nur77* and *Notch* is associated with T_RM_ cell maintenance [22,23]. The downregulation of *KLF2* is associated with *S1PR1* downregulation and tissue retention [24]. The downregulation of *Eomes* and *T-bet* is known to regulate the cytokine responsiveness of T_RM_ [25]. Hence, T_RM_ cells share a unique transcriptional program that is essential to tissue maintenance and function. Although it is not fully understood, the phenotypic and transcriptional characteristics of T_RM_ cells might be induced and maintained by antigen recognition and cytokine signaling such as interleukin-15 (IL-15) and transforming growth factor-β (TGF-β) [7,13], which might be responsible for the T_RM_ phenotype of most tumor-infiltrating T cells.

T_RM_ cells function against viral and bacterial infection more feasibly than T_CIRC_. They function not only as a primary defender against pathogens but also function as a facilitator; CD8+ T_RM_ can secrete various cytokines such as interferon-γ (IFN-γ), tumor necrosis factor, and interleukin-2 (IL-2), thereby triggering adaptive and innate immune responses rapidly including dendritic cell (DC) maturation, natural killer (NK) cell activation, and B cell recruitment [26]. This function of orchestrating immune responses is not only restrained to CD8+ T_RM_ cells but also reported in CD4+ T_RM_ cells, as they can trigger chemokine expression and magnify CD8+ T cell, NK cell, DC, and B cell responses upon antigen stimulation [27]. As a result of these properties, T_RM_ cells have been considered as an essential target of tumor immunotherapy.

## 3. Role of T_RM_ Cells in Anti-Tumor Immunity

The role of T_RM_ cells in anti-tumor immune responses is actively under investigation. The existence of TILs that express T_RM_ markers CD69 and CD103 has been reported in most human cancers. Intratumoral T_RM_ cells are distinct from the activated subset and upregulate genes associated with tissue residence, as shown in genomic profiling [28]. They are located in tumor border or intratumoral epithelial regions and possess better effector functions such as cytotoxicity and cytokine secretion compared with the non-T_RM_-like proportion of T cells [7].

### 3.1. CD8+ T_RM_ Cells

Recent evidence from ovary [29], breast [30], lung [31], liver [32], and bladder cancer [33] patients suggested that the enrichment of CD8+ T_RM_ cells correlates with the better survival. Moreover, the enrichment of CD8+ T_RM_ cells in lung [34], liver [32], breast [35], and laryngeal cancer [36] was associated with lower tumor stage. Therefore, T_RM_ cells might have a protective role in anti-tumor immunity, although there are few reports that implicated the regulatory or pro-tumorigenic role [8,37].

CD8+ T_RM_ cells are an important anti-tumor immune population not only in the established tumor but also in tumor surveillance before the tumor development. A recent study using a mice melanoma model clearly demonstrated that CD8+ T_RM_ cells have a critical role in the tumor surveillance by maintaining tumor-immune equilibrium [38]. In that study, tumor-specific T_RM_ cells surveyed melanoma cells, and T_RM_-deficient mice were more likely to develop tumors.

Although the recent study mentioned the CD8+ T_RM_ cell as a “sentinel” or “controller” against tumor, most studies have focused on their effector function as a “tumor killer”. Traditionally, CD8+ T_RM_ cells can exhibit an antigen-specific manner [39,40], but a recent report suggested that bystander CD8+ T cells are abundant in human tumor infiltrates, although their role in anti-tumor function needs to be elucidated [41]. Previous study showed that CD8+ TILs can directly kill autologous tumor in vitro [42]. In addition, a recent study has showed that CD103+ CD39+ CD8+ TILs kill autologous tumor cells in a MHC-class I-dependent manner [43]. Adhesion molecules expressed on CD8+ T_RM_ cells might facilitate their anti-tumor effector function. T cell infiltration and adhesion can be increased by CD103 [44]. The engagement of CD103 to E-cadherin expressed on tumor cells can enhance the signaling of effector program, resulting in increased granzyme B or IFN-γ [45,46]. In addition, CD49a has also known to facilitate anti-tumor function, because the blockade of CD49a decreased intratumoral T cell infiltration and tumor control [46,47,48]. Furthermore, CD8+ T_RM_ cells can act as “enhancer” of anti-tumor immunity, because it can perform antigen spreading through DCs [49]. In addition, interplay between the circulating CD8+ T cells and CD8+ T_RM_ cells within tumor tissues were helpful for the limiting tumor growth in mice [50]. These anti-tumor effects of CD8+ T_RM_ cells were validated in the parabiosis model, which showed that CD8+ T_RM_ cells do not recirculate in a preclinical murine tumor model, and this population enhanced the efficacy of cancer vaccine [51]. In summary, T_RM_ cells are a crucial population in protecting and killing the tumor cells, which are associated with the better prognosis of cancer patients.

### 3.2. CD4+ T_RM_ Cells

The characteristics and the role of CD4+ T_RM_ (non-Treg) cells in anti-tumor immunity is not well understood. Several previous studies uncovered that CD4+ T cells can help cytotoxic T lymphocyte (CTL) activity via the enhancement of migratory and invasive potential, differentiation, and survival, expansion of CTLs, or enhancement of CTL activity [52], which suggest that CD4+ T_RM_ cells might also have a beneficial role in the tumor killing. One recent study characterized the CD4+ T_RM_ cells in human lung cancer [53]. They expressed CD103, in addition to the CXCR6 and CD49a, and did not express T-bet and Eomes [53]. Targeting co-stimulatory receptors CD27 and CD28 to the CD4+ T_RM_ cells improved their cytokine secreting function [53]. However, the prognostic impact of CD4+ T_RM_ cells and the role in the ICI treatment for the cancers need to be more elucidated in future studies.

### 3.3. Tissue-Resident Treg Cells

Tissue-resident Treg cells are also important subset among CD4+ T cells in peripheral tissues. Treg cells express CD25 and FoxP3 and do not express CD127; hence, they are usually defined as CD25+ CD127−FoxP3+ CD4+ T cells. Treg cells target a broad range of immune cells: conventional CD4+ T cells, CD8+ T cells, macrophages, neutrophils, B cells, and DCs [54]. Soluble mediators from Treg cells such as interleukin-10 (IL-10), TGF-β, and adenosine have immunosuppressive effects. They also might have a lysing effect on the other immune cells [54]. Importantly, Treg cells in tumor tissue produce vascular endothelial growth factor (VEGF), which is associated with tumor angiogenesis and T cell exhaustion [55]. Tissue-resident Treg cells are well described in visceral adipose tissues [56,57], muscle [58], and skin [59]. They express distinct chemokine receptors such as CCR1, CCR2, and CCR9 have a unique T cell receptor (TCR) repertoire. Tumor-infiltrating Treg cells are enriched in various tumor tissues and associated with poor prognosis [60]. Importantly, tumor-infiltrating Treg cells express programmed death-ligand 1 (PD-L1) and programmed death-ligand 2 (PD-L2) [61]. In addition, they express LAYN, as well as CCR8-associated gene signatures, which is associated with poor prognosis [61]. Therefore, although the tissue-resident features of tumor-infiltrating Treg cells need to be more investigated, tumor-resident Treg cells should be considered as a target for the immunotherapy.

## 4. Role of T_RM_ Cells in ICI Treatment: Lessons from other Type of Cancers

T_RM_ cells are known to express various immune checkpoint molecules compared with the other subset of T cells; thereby, they should be considered as major target of ICI treatment. In addition, their property of long-term maintenance in peripheral tissues including tumors might be responsible for the long-term, durable responses to ICI treatment. Therefore, more understanding of the characteristics of T_RM_ cells in tumors might provide better clinical outcomes.

PD-1 is significantly more expressed on the CD8+ T_RM_ subset than the other subset of CD8+ T cells in normal peripheral tissues [62], which might not reflect an exhaustion feature of T cells. It is also expressed on CD8+ T_RM_ cells in tumors [29,42,63]. Although PD-1 might not be considered as a pure exhaustion marker in CD8+ T_RM_ cells, the fact is that CD8+ T_RM_ cells in tumors have less of an effector function compared with the CD8+ T_RM_ cells from surrounding non-tumor tissue, suggesting that they might respond to the ICI treatment, and their anti-tumor function might be enhanced. Anti-PD-1 treatment resulted in the expansion of intratumoral CD8+ T_RM_ cells in mice [50]. In addition, a previous study using TCR analysis suggested that intratumoral CD8+ T cells are proliferated by anti-PD-1 treatment in melanoma patients [64]. In vitro anti-PD-1 treatment enhanced the cytotoxic activity of CD8+ T_RM_ cells against autologous tumor cells in lung cancer patients [39].

In addition to the PD-1, intratumoral CD8+ T_RM_ cells also upregulate other immune checkpoint molecules such as T cell immunoglobulin and mucin-domain-containing-3 (TIM-3), lymphocyte-activation gene-3 (LAG-3), or cytotoxic T-lymphocyte-associated antigen-4 (CTLA-4) [28,31,35,65,66]. Among those various immune checkpoint molecules, CTLA-4 has been another representative therapeutic target for the various types of cancer, including HCC [67,68]. This molecule binds to the co-stimulatory molecules such as CD80 and CD86, and it competes with the CD28, thereby limiting the proper function of the T cells. Anti-CTLA-4 treatment was the firstly approved immunotherapy in human, and it has been used in melanoma patients actively, alone or in combination with anti-PD-1, which have a promising efficacy [68]. In addition, it is expressed on various intratumoral lymphocytes other than CD8+ T_RM_ cells such as Treg cells or non-Treg CD4+ T cells. Therefore, combination treatment targeting multiple immune checkpoint molecules might also provide better CD8+ T_RM_ responses, as shown in previous in vitro experiments [42,69,70]. In summary, these findings suggest that the multiple immune checkpoint molecules can suppress the effector function of CD8+ T_RM_, and targeting these molecules might be helpful for the enhancement of anti-tumor function of CD8+ T_RM_.

## 5. T_RM_ Cells in HCC

### 5.1. Characteristics of Tumor-Infiltrating CD8+ T Cells in HCC

**Figure 1:**
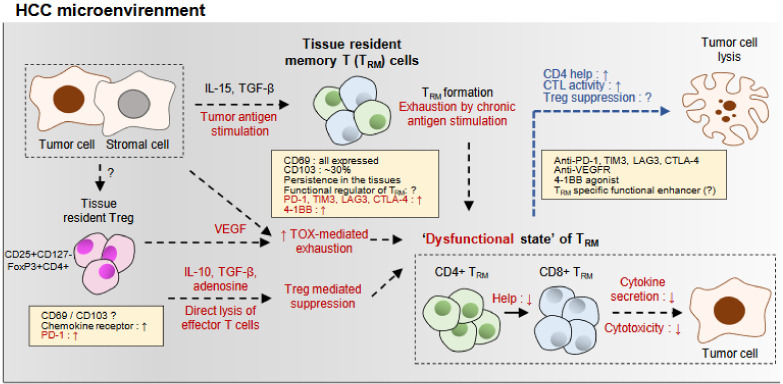
Tissue-resident memory T (T_RM_) cells in hepatocellular carcinoma (HCC). The summary of the generation, characteristics, and functional restoration of T_RM_ cells within HCC is presented. T_RM_ cells are generated by tumor antigen recognition in the presence of interluekin (IL)-15 and transforming growth factor-β (TGF-β) secreted by tumor cells and stromal cells such as fibroblasts. The mechanism of the generation of tissue-resident regulatory T (Treg) cells and their phenotypic characteristics are still not known. T_RM_ cells can persist within tumor tissues and perform tumor killing via cytokine secretion and cytotoxicity. However, due to the chronic antigen stimulation, vascular endothelial growth factor (VEGF, secreted by tumor cells, stromal cells, and Treg cells)-mediated enhancement of HMG-box transcription factor *TOX*-mediated T cell exhaustion and Treg-mediated suppression, T_RM_ cells enter a “dysfunctional state”, which is characterized by low CD4+ help, low cytokine secretion, and cytotoxicity against tumor cells. Therefore, targeting immune checkpoint molecules such as programmed cell death protein-1 (PD-1), T cell immunoglobulin and mucin-domain-containing-3 (TIM-3), lymphocyte-activation gene-3 (LAG-3), and cytotoxic T-lymphocyte-associated antigen-4 (CTLA-4) restores the dysfunction of the T_RM_ cells. In addition, because T_RM_ cells have unique phenotypic and transcriptional characteristics compared with other subset of T cells, whether targeting their own molecules might result in the enhancement of their anti-tumor function needs to be investigated. Each arrow indicate that the one before the arrow affects the one after it. Question mark represents the hypothesis which is unclear.

Recent technical advances facilitated the substantial studies investigating the immunology of HCC, including TILs. Two recent studies using the single-cell RNA sequencing of tumor-infiltrating T cells and immune cells from HCC have revealed their transcriptional characteristics [71,72]. Especially, this study revealed that exhausted CD8+ T cells and Treg cells are clonally expanded in tumors, and specific signature LAYN is associated with the suppressive feature of TILs [57]. These CD8-LAYN cells in HCC have highly expressed exhaustion markers such as *CTLA-4*, *PDCD1*, and *HAVCR2*, suggesting that they are exhausted cells by tumor-antigen stimulation [57]. Moreover, TCR sequencing revealed that HCC enriches clonal CD8+ T cells [57]. In another previous study, multiomic analysis integrating whole-exome sequencing, RNA sequencing, metabolomics, and proteomics was also conducted recently, and revealed three distinct subtypes of immunocompetent, immunodeficient, and immunosuppressive HCC, and T cell infiltration is different between those immune subtypes [73]. In addition, various chemokines and TGF-β were also found in the tumor tissues [73] (Figure 1), suggesting that the tumor microenvironment of HCC is also favorable to the existence of T_RM_ population.

### 5.2. Immune Checkpoint Molecules of Tumor-Infiltrating CD8+ T Cells in HCC

The detailed characterization of the exhaustive status of TILs of HCC in human and mice have been recently increasingly reported. Immune checkpoint molecules such as PD-1, TIM-3, LAG-3, and CTLA-4 on CD8+ T cells were significantly increased in HCC tissues compared with the control tissues and peripheral blood, which is associated with their impaired function [10,74,75,76] (Figure 1). Especially, they were expressed in the tumor-associated antigen (TAA)-specific CD8+ T cells [75]. Importantly, in vitro, antibodies against PD-L1, TIM-3, or LAG-3 enhanced the CD8+ T cell function such as proliferation and cytokine production, and combined treatment of PD-L1 with anti-TIM-3, LAG-3, or CTLA-4 further restored their function [75] (Figure 1). Thus, this study firstly provided the rationale for the combined ICI treatment for HCC using human HCC samples.

In addition, tumor-infiltrating CD8+ T cells in HCC had a distinct expression pattern of PD-1, which is characterized by PD-1-high, PD-1-intermediate, and PD-1-negative population, and this pattern was also associated with tumor aggressiveness [74]. In this study, PD-1-high CD8+ T cells expressed multiple immune checkpoint molecules, but responded more to the ICI treatment in vitro [74]. In addition, highly exhausted, PD-1-high CD8+ TILs could be further divided according to the expression of 4-1BB, which is co-stimulatory receptor, and 4-1BB-positive PD-1-high CD8+ TILs reflected more tumor-reactive and T cell activation [76]. Thus, the agonistic antibody to the 4-1BB in addition to the anti-PD-1 antibody further restored the CD8+ T cells in HCC [76] (Figure 1). These recent findings suggest that PD-1+ TILs, which might respond to the ICI treatment, are a heterogeneous population and need more to be characterized to reveal which subpopulation would respond more to the ICI treatment.

### 5.3. Molecular Mechanisms of T Cell Exhaustion of Tumor-Infiltrating CD8+ T Cells in HCC

Recently, TOX, which has a crucial role in T cell development and differentiation, has been reported as a promoter of CD8+ T cells in HCC [77] (Figure 1). In the presence of tumor antigen stimulation, TOX expression is increased in CD8+ T cells in HCC tissue, and this TOX upregulation is associated with the T cell dysfunction of CD8+ TILs [77]. In addition, TOX knockdown improved the response of anti-PD-1 treatment in vivo [77]. Importantly, TOX prevented the degradation of PD-1 via lysosome [77]. These findings suggest that TOX is the principal mediator of T cell exhaustion in HCC, and its applicability as a therapeutic target or a predictor for the treatment response or patients’ outcome should be validated in future studies.

Furthermore, dual blockade of the VEGF receptor-2 (VEGFR-2)-pathway and PD-1 inhibited tumor growth and survival in a murine orthotopic HCC model [78]. This study implicated that the dual blockade increased CD8+ T cell infiltration and activation, restored M1/M2 macrophage ration, and reduced Treg cells and CCR2+ monocytes in vivo [78]. This restoring effect of VEGFR blockade on the CD8+ T cells might be associated with the recent study that addressed that VEGF drives TOX-mediated T cell exhaustion in colorectal cancer [79] (Figure 1). In summary, these studies provide more insights in understanding the mechanisms and clinical implications of T cell exhaustion in HCC. Especially, targeting VEGFR in addition to the ICI treatment will be a hopeful strategy for the immunotherapy of HCC.

### 5.4. Tissue-Resident Phenotype of Tumor-Infiltrating CD8+ T Cells in HCC

However, there is lack of the studies that analyzed the HCC TILs in the context of T_RM_ cells as other cancers. One recent study described that the CD69+ population is the major population among TILs in HCC, although its CD103 positivity is about 20–30% [10] (Figure 1). In this study, the population of CD103-expressiong CD8+ TILs and PD-1-expressing CD8+ TILs is mostly overlapping. These findings suggest that T_RM_ cells in CD8+ TILS might be the main population that respond to the anti-PD-1 treatment. In addition, the exhausted population among CD8+ T cells that were investigated in previous studies can be regarded as the T_RM_ population. One previous study also observed the enrichment of CD8+ T_RM_ cells expressing CD69 and CD103 in HBV-related HCC, compared with the adjacent liver tissue and peripheral blood [32]. Interestingly, the frequency of CD8+ T_RM_ cells and PD-1 expression on the CD8+ T_RM_ cells were different between HBV-related HCC and non-viral HCC [32], suggesting that the etiology of HCC might have an impact on the generation or survival of CD8+ T_RM_ cells in HCC and thereby can affect the T cell immunity in HCC and the clinical outcome, especially receiving ICI treatment. Strikingly, this study demonstrated that the infiltration of CD8+ T_RM_ in tumors was associated with the favorable survival of HCC patients [32]. Therefore, future studies are needed to elucidate the detailed function, characteristics, and the role in the anti-tumor immunity of the T_RM_ population in HCC. They would provide the important clue to improve the outcome of ICI treatment in HCC.

### 5.5. CD4+ T_RM_ Cells in HCC

There are few previous studies that investigated tumor-infiltrating CD4+ T cells. A previous study reported that CD4+ T cells in HCC tissue also expressed multiple immune checkpoint molecules including PD-1, TIM-3, LAG-3, and CTLA-4 [75]. Furthermore, in vitro, antibodies against PD-L1, TIM-3, or LAG-3 enhanced the CD4+ T cell function such as proliferation and cytokine production, and combined treatment of PD-L1 with anti-TIM-3, LAG-3, or CTLA-4 further restored their function in response to the polyclonal stimulation or TAA [75] (Figure 1). In addition, under the anti-PD-1 treatment in addition to the anti-VEGFR-2, CD4+ T cells in HCC normalized vessel formation, which might be helpful for the anti-tumor effect of this regimen [78]. These findings suggest that tumor-infiltrating CD4+ T cells also can be a cellular target for the ICI treatment.

### 5.6. Tissue-Resident Treg Cells in HCC

Tumor-infiltrating Treg cells have been studied in human HCCs. Treg cells are increased in the peritumoral region of HCC [80] and intratumoral region [32,81], which was associated with CD8+ T cell impairment. Intratumoral Treg cells also inhibit the anti-tumor activity of γδ T cells [82]. Interestingly, the percentage of PD-1+ Treg cells in HCC was higher in HBV-related HCC and had more suppressive activity, which was diminished by anti-PD1/anti-PD-L1 blockade [32]. Strikingly, these intratumoral Treg cells were associated with poor patient outcome [32]. In the recent report, FoxP3 expression in tumor-infiltrating CD4+ T cells is observed to overlap with the CD69 expression, suggesting that tumor-infiltrating Treg cells are a tissue-resident population [10] (Figure 1). Furthermore, this population seemed to mostly express PD-1 [10]. In addition, intratumoral Treg cells can produce VEGF, which is associated with tumor angiogenesis [55] and TOX-mediated T cell exhaustion [79] in other cancers. Therefore, tissue-resident Treg cells in HCC might also be an important target for the ICI treatment and VEGF blockade in HCC.

Nevertheless, these previous studies do not clearly delineate the tissue-resident and non-resident population. Since most intratumoral CD4+ T cells contain CD69+ cells [10], HCC might also contain the CD4+ T_RM_ population. Thus, both conventional CD4+ T_RM_ cells and tissue-resident Treg cells need to be investigated in future studies.

## 6. Characteristics of Liver T_RM_ Cells: Implication to Tumor Immunosurveillance

As previously discussed in the prior section, T_RM_ cells play a role in the surveillance against tumor cells [38]; thereby, they inhibit tumor development and growth in skin. However, the role of liver T_RM_ cells in the HCC surveillance needs to be elucidated.

**Figure 2:**
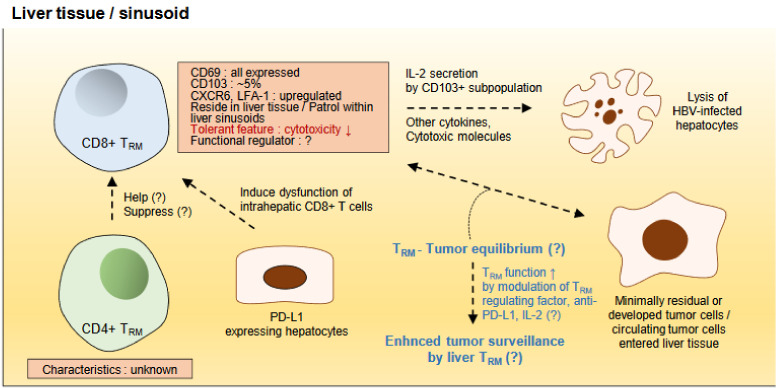
Suggestive model of tumor surveillance by liver T_RM_ cells. The liver CD8+ T_RM_ cells have been studied in several previous reports. They express CD69, but CD103 is expressed in the small portion among the CD69+ population in human. They express chemokine receptor type 6 (CXCR6) and lymphocyte function-associated antigen-1 (LFA-1) and persist in the liver tissue or liver sinusoid. Although their regulating factor is still unknown, liver CD8+ T_RM_ cells are considered to be functionally impaired or tolerant compared with the other T cell population. This might be associated with the priming by PD-L1-expressing hepatocytes. The CD103+ subpopulation is known to perform an anti-viral function, because they can highly secrete IL-2 and other effector molecules against hepatitis B virus (HBV) peptide stimulation. However, the characteristics of CD4+ T_RM_ cells and their role in the tumor surveillance are still unknown. T_RM_-tumor equilibrium (left-right arrow) has not been studied in the HCC model, but as in other cancers, liver CD8+ T_RM_ cells might have a role in the surveillance against minimally residual tumor cells after HCC treatment, newly developed tumor cells, or circulating tumor cells that enter the liver tissue or sinusoid. This function of liver CD8+ T_RM_ cells could be enhanced by anti-PD-L1 or IL-2 treatment according to the previous report of murine skin cancer model or modulation of regulating factors of liver CD8+ T_RM_ cells, which needs to be elucidated in future studies. Each leftwards or rightwards arrow indicate that the one before the arrow affects the one after it. Question mark represents the hypothesis which is unclear.

Liver CD8+ T_RM_ cells, but not liver CD4+ T_RM_ cells in mice and human, have been recently reported. Liver T_RM_ cells performed front-line defense against liver-stage malaria in mice [14]. That study firstly found that murine liver CD8+ T_RM_ cells express CD69 but do not express CD103 and mainly patrol within the sinusoids. Another study found that lymphocyte function-associated antigen-1 is expressed on liver CD8+ T_RM_ cells, which is essential for the patrolling and persistence in the liver sinusoids [83] (Figure 2). Interestingly, human liver CD8^+^ T_RM_ cells had low cytolytic capacity in terms of ex vivo expression of perforin and granzyme B [84], suggesting that they could contribute to the immune-tolerant niche of liver environment (Figure 2). On the other hand, a small portion of CD69+ CD8+ T_RM_ cells contains CD103+ cells in human, and they mainly secrete IL-2 and highly activated by HBV-antigen stimulation [15], suggesting that the characteristics of CD8+ T_RM_ cells in human and mouse can be different (Figure 2). A recent elegant study has suggested that liver CD8+ T cells primed by hepatocytes show dysfunctional features, which cannot be rescued by anti-PD-L1 but by IL-2 [85] (Figure 2).

These previous studies suggest that liver CD8+ T_RM_ cells make up a unique population that has low cytotoxic capacity, which is in line with the immune-tolerant niche of the liver. Since this dysfunction might not be restored by anti-PD-L1 or anti-PD-1 alone, additional strategies including cytokines such as IL-2 might be synergistic in boosting the anti-tumor immunity of liver CD8+ T_RM_ cells surrounding HCC (Figure 2). Moreover, this strategy also need to be considered in the adjuvant setting of immunotherapy for the HCC to enhance the surveillance of CD8+ T_RM_ cells against minimally remaining or newly developing tumors. In addition, the characteristics of CD4+ T_RM_ cells in the liver also need to be elucidated.

## 7. Unconventional, Innate-Like Resident T Cell Population in HCC

**Figure 3:**
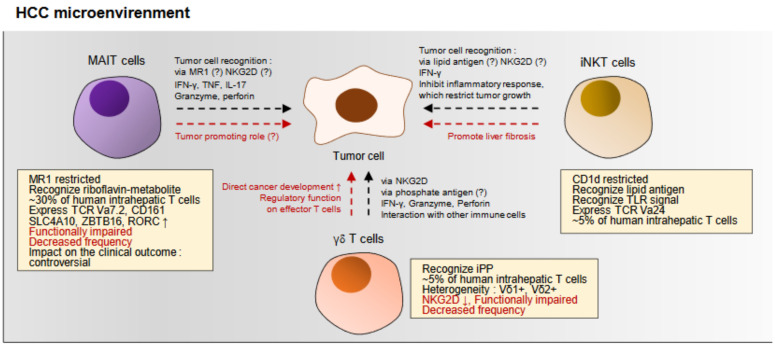
Unconventional, innate-like T cells in HCC. Unconventional T cells such as mucosal associated invariant T (MAIT) cells, γδ T cells, and invariant natural killer T (iNKT) cells consist ≈40% of liver resident T cells. However, their role in the anti-tumor immunity in HCC needs to be elucidated in future studies. MAIT cells are a major population of intrahepatic and HCC-infiltrating lymphocytes. Although the tumor recognition mechanism is unclear, they secrete cytokines and perform cytotoxicity against tumor cells. They are functionally impaired and reduced in the HCC. Impact on the patient survival of the MAIT cells is a controversial issue, because two previous studies reported different results. The tumor-promoting role of the MAIT cells is unclear. γδ T cells also exist in the HCC, although their frequency is decreased and they are functionally impaired. There are reports that γδ T cells have a tumor-killing effect via cytokines and cytotoxic activity or interaction with other immune cell population such as B cells, natural kilter (NK) cells, or conventional T cells. On the other hand, there are also previous reports that suggested the tumor promoting role of these cells. This might be due to the heterogeneity of γδ T cells. iNKT cells also have anti-tumor role via cytokine secretion or inhibiting oncogenic, inflammatory signals in the liver. However, they also can promote liver fibrosis. Therefore, the role of unconventional, innate-like T cells in HCC needs to be investigated in future studies. Each arrow indicate that the one before the arrow affects the one after it. Question mark represents the hypothesis which is unclear.

Among liver tissue-infiltrating lymphocytes, the unconventional, innate-like T cell population consists 20%–50% of CD3+ T cells. MAIT cells, γδ T cells, and invariant NKT (iNKT) cells are a representative population of unconventional T cell population in the liver (Figure 3). They use very restricted TCR repertoire and do not stimulated through the conventional TCR-MHC-mediated activation pathway. In addition, they mostly express CD69, although their CD103 expression and tissue-resident phenotypes need to be elucidated in future studies. In addition, the role of these populations in the anti-tumor immunity, especially in the HCC is not well understood.

### 7.1. MAIT Cells

MAIT cells are unconventional T cells, which recognize MHC class I-like molecule, MR1-loaded non-peptide antigens, mainly metabolites from the rivoflavin biosynthesis pathway in some species of bacteria such as 5-(2-oxopropylideneamino)-6-D-ribitylaminouracil (5-OP-RU) [86]. They consist ≈30% of human intrahepatic T cells; thus, they are major population of the human liver T cells but not in mice (≈5%). They all express TCR Vα7.2 joined to the TCR Jα33 and mostly express CD161, although they tend to downregulate in some environments such as tumor tissues [86]. Primarily, they provide protective immunity against bacterial infection, but they also play a positive or negative role in the autoimmune, inflammatory, and metabolic diseases via a TCR-independent manner [87]. However, their role in the anti-tumor immunity is still controversial and not well understood. Especially, how they can recognize tumor cells (e.g., MR1 dependent or independent) is unsolved, but it is an important question of this field.

A recent study using single-cell RNA sequencing of HCC-infiltrating T cells revealed that MAIT cells are distinctly clustered and consist of a considerable portion among TILs [71]. These cells are characterized by the expression of *SLC4A10*, *ZBTB16*, and *RORC* and use semi-invariant TCR alpha chains [71]. In addition, this study observed a marked decrease of MAIT cells in HCC tissues compared with the adjacent tissues, and the decrease of MAIT cells was associated with the poor survival of the patients [71] (Figure 3). These findings suggest that MAIT cells have a beneficial role in the anti-tumor immunity and might have a tumor-killing effect in HCC (Figure 3). On the other hand, another recent report suggested that MAIT cells are depleted, activated, and exhausted in HCC, and the enrichment of MAIT cells are associated with the poor outcome of HCC patients [88] (Figure 3). Therefore, further study is needed to determine whether MAIT cells have an anti-tumor effect or a tumor-promoting effect in HCC.

### 7.2. γδ T Cells

γδ T cells, which have γ and δ chain TCRs, consist ≈10% of human intrahepatic T cells. To lyse target cells, they use NKG2D and TCR with the ligands MHC class I chain-related molecule A/B and isopentenyl pyrophosphate [89] and play a role in the anti-microbial function. Their role in the anti-tumor immunity is controversial. They can perform a direct anti-tumor activity and indirect anti-tumor activity via interacting with B cells, DCs, conventional T cells, and NK cells [90]. However, they also can directly promote cancer development, restrict other T cell function, and enhance the function of regulatory immune cells such as myeloid-derived suppressor cells [90]. This might be due to the their heterogeneity, because there are Vδ1+, Vδ2+, or non-Vδ1Vδ2 subsets in the total γδ T cells, and their characteristics are very different, although those are not to be discussed in this review. There is one previous report that observed the γδ T cells in HCC. In this study, γδ T cells were significantly decreased in the HCC tissues, and they were also functionally impaired in terms of the cytotoxicity and cytokine secretion [82] (Figure 3). This functional impairment was associated with Treg-derived TGF-β and IL-10 [82]. In summary, γδ T cells might play a role in the anti-tumor immunity in HCC, but detailed characterization according to the various subsets of γδ T cells in HCC tissues is needed.

### 7.3. iNKT Cells

iNKT cells are an abundant population among murine liver T cells (≈30%) but relatively small population among human liver T cells (≈5%). They generally are restricted to the CD1d and recognize microbial lipid antigens by signaling through Toll-like receptors [91]. They use a very restricted TCR repertoire and express TCR Vα24. Upon activation, they can secrete cytokines and perform cytotoxicity as another unconventional, innate-like T cell population [91]. iNKT cells also have a dual role in anti-tumor immunity. They can activate conventional CD8+ T cell responses, but they can also be associated with tumor growth via recruiting Treg cells and Th2 cytokines, which might be associated with restriction of the expansion of TAA-specific CD8+ T cells [92].

iNKT cells have a tumor-suppressive function in HCC, because a previous study implicated that oncogenic beta-catenin triggers an inflammatory response, but iNKT cells inhibited this inflammatory responses, thereby inhibiting murine HCC growth in vivo [93] (Figure 3). Importantly, a recent study showed that alteration of the gut microbiome can affect the function of iNKT cells, which restrict growth of HCC [94] (Figure 3). However, the role in the anti-tumor immunity, especially their mechanism of the tumor-inhibiting effect, needs to be more elucidated. In addition, they are also associated with liver fibrosis [95,96,97] (Figure 3), which might be associated with HCC development.

## 8. Concluding Remarks

As discussed, tumor-resident lymphocytes in HCC and liver-resident lymphocytes have crucial roles in anti-tumor immunity. It is well known that the liver is an immunological organ, and various and abundant lymphocytes reside in the liver sinusoids and liver tissues. Therefore, they should be considered as a primary target of immunotherapy for the HCC. Since they have distinct phenotypes, transcriptional programs, and developmental programs, they need to be more characterized; therefore, we need to seek complementary strategies to stimulate this population. For example, because various types of tissue-resident lymphocytes express CTLA-4, anti-CTLA-4 treatment might be a candidate of another immunotherapeutic regimen, and several human trials are undergoing using this anti-CTLA-4 in combination with anti-PD-1 [67]. Furthermore, anti-PD-1 (or PD-L1) plus anti-VEGFR blockade would be a promising combination, because they can both target cytotoxic CD8+ T_RM_ cells and tissue-resident Treg cells. Indeed, a recent clinical trial was successful, which used atezolizumab plus bevacizumab for unresectable HCC [98]. This study showed this combination regimen of anti-PD-L1 plus anti-VEGF had significantly longer overall and progression-free survival compared to the sorafenib, which was the first successful regimen as a first-line treatment for the unresectable HCC beyond sorafenib. As discussed in the previous sections, T_RM_ cells might be front-line responders of this combination regimen. The detailed characterization of T_RM_ cells might allow finding additional molecular targets for the immunotherapy of HCC. Furthermore, the biomarkers for the ICIs are still need to be elucidated despite the promising results of this combination regimen, and T_RM_ cells in HCC patients might be one of the candidates.

An analysis for the tissue-resident lymphocytes before and after ICI treatment would be also important, because they are the primary tumor-killing population and ICI-responding population. In addition, which subset of tissue-resident lymphocytes would better respond to the ICI treatment would be important for developing future biomarkers of ICI responsiveness. Furthermore, better analysis of these cells would provide helpful insights for the adoptive cell therapy for HCC, because they have the ability to persist in the peripheral tissues and respond to the antigens rapidly. Finally, whether loco-regional treatment such as TACE and radiofrequency ablation can modulate tissue-resident lymphocytes and can be combined with ICI treatment would be also interesting issues. These precision and individualized approaches need to be validated in future well-designed clinical trials. One of the good examples is a recent human, which suggested that HCC patients with PD-1high CD8+ TILs had aggressive tumor features but responded well to the anti-PD-1 and another ICI treatment in vitro [74].

Nevertheless, obtaining liver samples from patients with HCC has been one of the hurdles for monitoring the liver-resident immune cell population. Therefore, in addition to the tissues from hepatectomy and liver core needle biopsy, or perfusates from LT, fine needle aspiration, which is a relatively easy and safe technique, is being tried recently for the immune monitoring and profiling in patients with chronic HBV infection, and it might be also feasible in the patients with HCC.

In conclusion, tissue-resident lymphocytes are a liver-abundant population and also can be found in HCC tissues. Their contribution in anti-tumor immunity against HCC varies among subpopulations, but future studies regarding this population would improve the outcome of HCC.

## Data Availability

Not applicable.
